# Dietary Essential Amino Acids Affect the Reproduction of the Keystone Herbivore *Daphnia pulex*

**DOI:** 10.1371/journal.pone.0028498

**Published:** 2011-12-05

**Authors:** Patrick Fink, Claudia Pflitsch, Kay Marin

**Affiliations:** 1 Cologne Biocenter, Department of Aquatic Chemical Ecology, University of Cologne, Cologne, Germany; 2 Institute of Biochemistry, University of Cologne, Cologne, Germany; Institute of Marine Research, Norway

## Abstract

Recent studies have indicated that nitrogen availability can be an important determinant of primary production in freshwater lakes and that herbivore growth can be limited by low dietary nitrogen availability. Furthermore, a lack of specific essential nitrogenous biochemicals (such as essential amino acids) might be another important constraint on the fitness of consumers. This might be of particular importance for cladoceran zooplankton, which can switch between two alternative reproductive strategies – the production of subitaneously developing and resting eggs. Here, we hypothesize that both the somatic growth and the type of reproduction of the aquatic keystone herbivore *Daphnia* is limited by the availability of specific essential amino acids in the diet. In laboratory experiments, we investigated this hypothesis by feeding a high quality phytoplankton organism (*Cryptomonas*) and a green alga of moderate nutritional quality (*Chlamydomonas*) to a clone of *Daphnia pulex* with and without the addition of essential amino acids. The somatic growth of *D. pulex* differed between the algae of different nutritional quality, but not dependent on the addition of dissolved amino acids. However, in reproduction experiments, where moderate crowding conditions at saturating food quantities were applied, addition of the essential amino acids arginine and histidine (but not lysine and threonine) increased the total number and the developmental stage of subitaneous eggs. While *D. pulex* did not produce resting eggs on *Cryptomonas*, relatively high numbers of resting eggs were released on *Chlamydomonas*. When arginine and histidine were added to the green algal diet, the production of resting eggs was effectively suppressed. This demonstrates the high, but previously overlooked importance of single essential amino acids for the reproductive strategy of the aquatic keystone herbivore *Daphnia*.

## Introduction

In many classes of organisms, survival during periods of unfavourable environmental conditions is facilitated by the production of resting stages [1]. Resting stages are used as a dispersal strategy, particularly by aquatic and stenoecious organisms, since the desiccated resting stages can bridge large distances between favourable habitats [2]. In most crustaceans, subitaneous reproduction occurs at relatively high per-capita rates, whereas only a few resting stages per capita are produced. Hence, the switching from subitaneous reproduction to the production of resting stages is associated with high demographic costs as the high population growth rates possible through the production of subitaneous eggs can not be maintained [1]. Minimizing this trade-off is of particular importance for cladoceran zooplankton, since they are common in temperate ecosystems with strong seasonal variations in environmental parameters and even temporary ponds which desiccate for parts of the season. The optimal timing of this switch to diapause induction is thus probably under strong selection [3]. During most of the season, the common cladoceran genus *Daphnia* produce parthenogenetic subitaneous eggs which immediately develop within the mothers' brood pouch and are released with the next maternal molt cycle. The neonates hatching from these subitaneous eggs normally produce subitaneous clutches as well. By this cyclic parthenogenesis, daphnids reach high population growth rates, and this is considered to be one of the keys to their immense success and dominance in many pond and lake ecosystems worldwide [4]. Only when environmental conditions deteriorate, *Daphnia* undergo a diapause by producing resting eggs enclosed in a protective capsule (ephippium). A multitude of environmental factors have been described to induce resting egg formation, ranging from photoperiod to food availability and population density (crowding, [5], [6]) and to infochemicals that indicate the presence of predators [7], [8]. Some of these triggers even have to act simultaneously to induce resting egg formation in some genotypes of *Daphnia* [8], [9]. Usually, resting egg production in *Daphnia* is a sexual reproduction, which requires a production of male offspring and a meiosis in the ovogenesis [10]. This allows genetic recombination and helps to maintain the genetic diversity of the population. Some clones of the *Daphnia pulex* species group have lost the ability for sexual reproduction and produce asexual resting eggs which are genetically identical to their mothers [11], [12]. These clones do not produce males, thus saving the cost of producing males [13].

The switch between the two reproductive strategies (production of either subitaneous or resting eggs) is under hormonal control [14], [15], and in most temperate lake ecosystems, production of resting eggs is limited to relatively distinct periods in early summer and autumn [16]. In these times of the season, the plankton community undergoes drastic changes driven by external (light, temperature) and internal (grazing, competition) factors. Since daphnids as filter-feeders unselectively retain food items of a broad size range (approx. 1–55 µm) on the filter screens of their thoracic appendages, a discrimination based on the nutritional quality of individual food items is not possible and thus such quality differences can directly impact growth and reproduction of *Daphnia*. Considering the high requirements for energy and essential nutrients for resting egg production by *Daphnia* [1], [17], [18], surprisingly little research has focussed on the steering role of food quality for the reproductive strategies of daphnids. Among the biochemical constituents of phytoplankton known to influence the nutritional quality of primary producers for daphnids are essential polyunsaturated fatty acids (e.g., [19], [20]) and sterols (e.g., [21]). Further, the the dietary availability of essential amino acids (EAA) might potentially constrain the fitness of herbivores across ecosystems, independent of the total nitrogen availability, although quantitative data on this hypothesis is scarce [22]. While earlier studies described the induction of resting stages to be dependent on food quantity (e.g., [23]), we here focus on the role of food quality, i.e. the diet's content in essential nutrients. Except for the clear-water phase, seston quantities are often sufficiently high for *Daphnia* in temperate lakes [20], which highlights the potential importance of limitations of zooplankton production by food quality [24]. Such a food quality effect on the production of resting stages has recently been described for the essential polyunsaturated eicosapentaenoic acid in a clone of *Daphnia pulicaria* [17]. Only recently, Koch et al. [3] demonstrated that addition of protein beads to a green algal diet enhanced the population growth rate of *D. pulex* and *D. galeata* by suppressing the production of resting eggs. These results suggested that either protein limitation per se or limitation by a certain constituent of this protein (e.g., EAA) can induce *Daphnia* resting egg production. Here, we hypothesize that the ephippia-suppressing effect of protein beads in the study of Koch et al. [3] is caused by specific essential amino acids. For *Daphnia*, the same amino acids are considered to be essential as for insects and mammals [25], and the pool of free amino acids in *D. magna* is dominated by histidine, arginine, alanine, and glutamate [26]. Among those, the two essential amino acids arginine and histidine were shown to be particularly important for growth of marine decapods [27], [28]. Therefore, we hypothesized that the availability of dietary EAA in general and arginine and histidine specifically, would affect both the choice of reproductive strategy in *Daphnia*, and also the somatic growth of this herbivorous zooplankton. To investigate these hypotheses, we conducted controlled growth and reproduction experiments with a clone of *Daphnia pulex* and – similar to the study of Koch et al. [3] – two phytoplankton species of known food quality difference. In these experiments, we added dissolved EAAs to the experimental units to investigate whether a diet enriched in EAAs (in particular arginine and histidine) would enhance the somatic growth and affect the reproductive strategy of *D. pulex* on the low quality green algal diet (*Chlamydomonas* sp.), but not on the high quality cryptophyte diet (*Cryptomonas* sp.). To investigate whether the effect of arginine and histidine would be a specific one or a general effect of EAA addition, the EAAs lysine and threonine were also supplied to *D. pulex* in some of the experiments.

## Methods

### Cultures

Pure (unialgal, but not axenic) cultures of *Cryptomonas* sp. (Ehrenberg 1838) strain SAG 26.80 and of *Chlamydomonas klinobasis/globosa* (Skuja 1956) strain #56 were obtained from the Sammlung für Algenkulturen, Göttingen, Germany and the Culture Collection at the Limnological Institute of the University of Constance, Germany, respectively. In the following, the genus names will be used to abbreviate the food organisms. Both algae were cultivated semicontinuously as suspension cultures at a dilution rate of 0.25 d^–1^ in Cyano medium [29] with added vitamins (Thiamine Hydrochloride 0.3 µM, Biotin 0.002 µM, and Cyanocobalamine (Vit. B12) 0.004 µM). An obligately parthenogenetic clone (i. e. one that asexually produces diploid resting eggs that are genetically identical to the mothers) of *Daphnia pulex* (Leydig 1860) was used for the experiments. Daphnids were cultivated in filtered (0.45 µm) aged tap water at 20°C under constant dim light and fed algae (*Chlamydomonas* or *Cryptomonas*, respectively) equivalent to 2 mg particulate organic carbon (POC) L^−1^.

### Growth experiments

For the standardized growth experiments, third brood juveniles of *D. pulex* (pre-cultured with *Chlamydomonas*) were collected within 12 h of birth and placed in 1.5 L jars with 1 L of filtered and aged tap water each. Jars were replicated threefold and placed in a climate-controlled chamber at 20°C and constant dim light. Food suspensions (*Chlamydomonas* or *Cryptomonas*, respectively, equivalent to 2 mg POC L^−1^) were renewed daily to avoid a depletion of the food quantity below the incipient limiting level. For the following six days, the animals were daily transferred into fresh water with the respective food algae. The essential amino acids arginine (Arg), histidine (His), lysine (Lys) and threonine (Thr) were obtained as pure compounds (analytical standards, all ≥ 98% purity) and dissolved in ultrapure water to yield 100 µM stock solutions. Arg and His were chosen because of their known effects for the growth of marine decapods [27], [28], Lys and Thr were two arbitrarily chosen EAA to test whether possible effects would be specific to Arg and His or rather general effects of EAA addition. These amino acid solutions were added to the respective experimental treatments to yield final concentrations of 25 µM of the respective EAA. The somatic growth rate of the juvenile daphnids was calculated according to [19] as *g  =  (ln (W_t_) – ln (W_0_))/t*, where W is the body dry weight of a subsample of the experimental animals at the beginning (*W_0_*) and the end (*W_t_*) of the experiment and *t* is the duration of the experiment in days. Subsamples for dry weight comprised 15 individuals at *W_0_* and 9-10 individuals at *W_t_* with n = 3.

### Reproduction experiments

To investigate how the availability of EAA affects the reproductive strategy of *D. pulex*, we performed reproduction experiments. In these experiments, *D. pulex* was supplied with *Chlamydomonas* or *Cryptomonas* in combination with dissolved EAA. To induce the production of resting eggs, *D. pulex* was cultivated under moderate crowding conditions, which is known to be a strong ephippia-inducing factor in *D. pulex* [5]. Other ephippia-inducing factors such as photoperiod or food shortage [9] were deliberately excluded. 10–15 third-brood neonates of *D. pulex* were placed into 200 ml jars with 0.2 µm membrane-filtered, aged tap water. During the experiments, the number of individuals per jar was reduced to 6-10 (equivalent to 30-50 ind L^−1^) to avoid a limitation by the food quantity. In the first reproduction experiment, *D. pulex* was fed either *Chlamydomonas* or *Cryptomonas* (with addition of either Arg and His, Lys and Thr or no additional EAA) as food organisms in saturating quantities (2 mg POC L^−1^). In the second reproduction experiment, *D. pulex* was fed either *Chlamydomonas* (with addition of either Arg, His, both Arg and His or no additional EAA) or *Cryptomonas* without additional EAA. In the EAA supplementation treatments, the respective EAA were added as dissolved EAA (25 µM final concentration) as described above. If multiple EAA were added, each was added at a final concentration of 12.5 µM to yield a total EAA concentration of 25 µM even in treatments with two added EAAs. Individuals were transferred daily into fresh medium (0.2 µm membrane-filtered, aged tap water with 2 mg POC L^−1^ of the respective food organism) and the number of subitaneous neonates and shed ephippia from the first three clutches were recorded. The experiments lasted for 12–15 days until all experimental animals had either produced three subitaneous clutches or two clutches with resting eggs (which takes longer) and the number of released subitaneous neonates and resting eggs per female and day were counted.

In a third reproduction experiment, we investigated whether the availability of EAA would also influence the development of subitaneous eggs in the maternal brood chamber. In a setup similar to the growth experiments described above, synchronized third clutch neonates of *D. pulex* were fed either *Cryptomonas*, *Chlamydomonas*, *Chlamydomonas* with Arg and His or *Chlamydomonas* with Lys and Thr for six days (10 individuals per jar with n = 4). At the end of this period, the animals had deposited their first clutch of eggs in their brood chamber. Because the animals were sampled for dry mass at the same time-point for the determination of somatic growth rates, we could not wait until they released their first clutch, which would have allowed us to determine the age at first reproduction. Instead, prior to drying, the animals were anesthetized with carbonated water and the egg developmental stages in the daphnids' brood chambers classified according to Threlkeld [30].

### Amino acid analyses

To analyze the cellular content of the two EAA Arg and His in cultures of *Chlamydomonas* and *Cryptomonas*, algal biomass was harvested from exponentially growing cultures (see above) by centrifugation at 2000 x g. After removal of the supernatant, pellets (equivalent to 50–200 µg POC) were resuspended with 20% perchloric acid (Merck) and 500 µM of taurine as internal standard. Subsequently, samples were hydrolyzed at 110°C for 24 h. After neutralization and precipitation of potassium perchlorate by addition of 5 M KOH with 1 M triethanolamine, the samples were centrifuged at 13.000 x g and the supernatant subjected to HPLC analysis. Amino acids were measured fluorimetrically after precolumn derivatization with o-phtaldialdehyde reagent (Pierce, Oud-Beijerland, The Netherlands) and separated on a reversed-phase column (Merck Lichrospher RP-18, 1503 mm) using a Hitachi Elite LaChrome HPLC system [31]. Quantification of Arg and His in triplicate algal samples (µmol amino acid mg POC^−1^) was based on calibrations for each amino acid and calculated using the peak areas of the respective amino acids and the internal standard.

### Statistical analyses

All data were tested for homoscedasticity prior to comparing the somatic growth rates from the growth experiments and the numbers of subitaneous eggs from the reproduction experiments using an analysis of variance (ANOVA) followed by post-hoc Tukey HSD tests with the GLM module of Statistica v.6 software package (StatSoft Inc.). Since the numbers of resting eggs in the first reproduction experiment contained zero values, this data was analyzed using a Kruskal-Wallis ANOVA followed by pairwise comparisons with Mann-Whitney U-tests from the nonparametrics module of Statistica. The algal amino acid contents and the data on the egg developmental stages were compared via one-way ANOVA using SigmaPlot v.11 (Systat Software Inc.). The significance level for all statistical analyses was α = 0.05.

## Results

### Growth experiments

**Figure 1:**
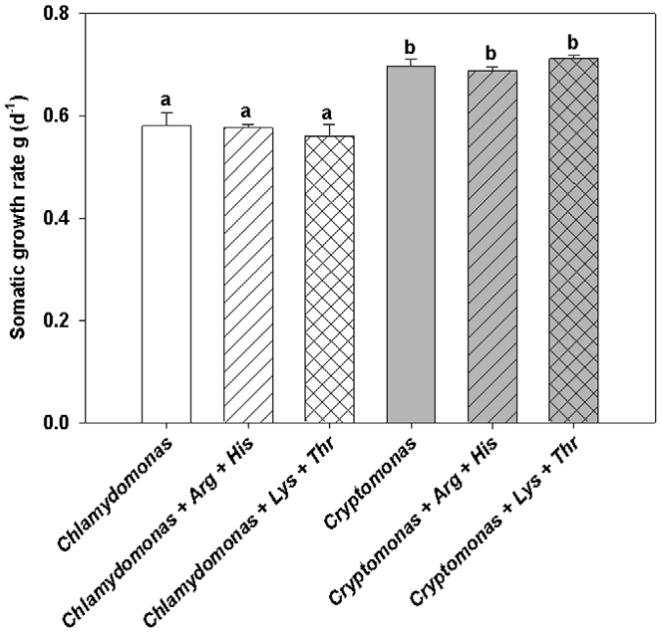
Growth on algae with and without EAA addition. Somatic growth rates (mean of n = 3 ± SD) of *D. pulex* fed *Chlamydomonas* or *Cryptomonas*, both either without additional EAA or with the addition of dissolved arginine and histidine or lysine and threonine at concentrations of 25 µmol L^−1^; identical lowercase letters indicate no significant difference after Tukey's post-hoc HSD test following one-way ANOVA.

While there was a significant effect of the food alga on the somatic growth rate of *D. pulex* (one-way ANOVA F_5,18_ = 78,08; p<0,001) with *Cryptomonas* being of superior quality compared to *Chlamydomonas*, there was no significant effect of the addition of EAA (neither Arg and His, nor Lys and Thr) on the somatic growth of *D. pulex* (Fig. 1).

### Reproduction experiments

**Figure 2:**
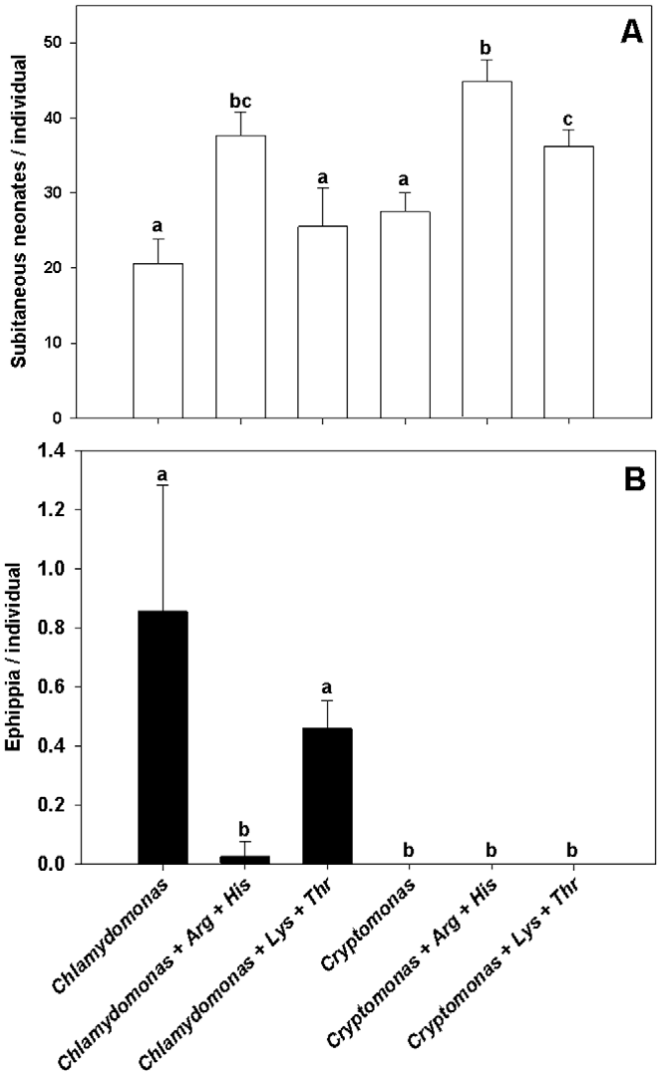
Reproduction on algae with and without EAA addition – exp. 1. Cumulative production of subitaneous eggs (A) and ephippia (resting eggs, B) by *D. pulex* fed *Chlamydomonas* or *Cryptomonas*, both either without additional EAA or with the addition of dissolved arginine and histidine or lysine and threonine at concentrations of 25 µmol L^−1^; egg numbers are means of n = 3 ± SD; identical lowercase letters indicate no significant difference after Tukey's post-hoc HSD test following one-way ANOVA for the subitaneous eggs and Mann-Whitney U-tests following Kruskal-Wallis ANOVA for the resting eggs.

In the first reproduction experiment, both the green alga *Chlamydomonas* and the Cryptophyte *Cryptomonas* were fed to *D. pulex* with and without the addition of either dissolved Arg and His or Lys and Thr. A one-way ANOVA demonstrated highly significant differences between treatments for the number of subitaneous eggs (F_5,18_ = 30.023, p<0.0001, Fig. 2A). A Kruskal-Wallis ANOVA on the number of produced resting eggs revealed highly significant between-treatment differences as well (H_5,24_ = 20.856, p<0.001, Fig. 2B). When fed a diet consisting of *Chlamydomonas* with or without the addition of Lys and Thr, *D. pulex* frequently produced clutches of resting eggs instead of subitaneous eggs (Fig. 2). Addition of Arg and His to a diet of *Chlamydomonas* effectively suppressed the resting egg production of *D. pulex* (Fig. 2): No ephippia production but a high number of subitaneous neonates was observed in this treatment, similar to the treatments with *Cryptomonas* as the food organism. Upon the addition of Arg and His to either of the two food algae, the number of subitaneous eggs increased, while the addition of Lys and Thr only caused a significant increase in the number of subitaneous eggs on *Cryptomonas*, but not on *Chlamydomonas* as food organism of *D. pulex* (Fig. 2).

**Figure 3:**
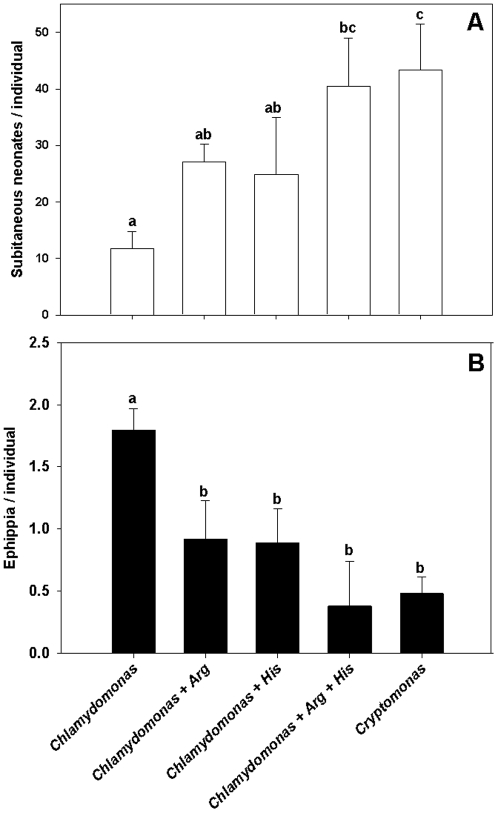
Reproduction on algae with and without EAA addition – exp. 2. Cumulative production of subitaneous eggs (A) and ephippia (resting eggs, B) by *D. pulex* fed either *Cryptomonas*, *Chlamydomonas, Chlamydomonas* with the addition of dissolved arginine, histidine or both arginine and histidine at concentrations of 25 µmol L^−1^; egg numbers are means of n = 3 ± SD; identical lowercase letters indicate no significant difference after Tukey's post-hoc HSD test following one-way ANOVA.

In the second reproduction experiment, *Chlamydomonas* was fed to *D. pulex* with and without the addition of only Arg, His or a combination of both amino acids. Furthermore, *Cryptomonas* without additional amino acids was offered as control food in another treatment. Similar to the first reproduction experiment, *D. pulex* produced resting eggs at high numbers when fed *Chlamydomonas* alone. Both feeding with *Cryptomonas* and addition of Arg and/or His suppressed the resting egg formation and increased the number of subitaneous offspring (Fig. 3). Again, the comparison of the number of produced subitaneous and resting eggs via one-way analysis of variance and Tukey's HSD showed highly significant differences between treatments both for resting eggs (F_4,15_ = 17.826, p<0.0001) and subitaneous eggs (F_4,15_ = 12.639, p<0.001).

**Figure 4:**
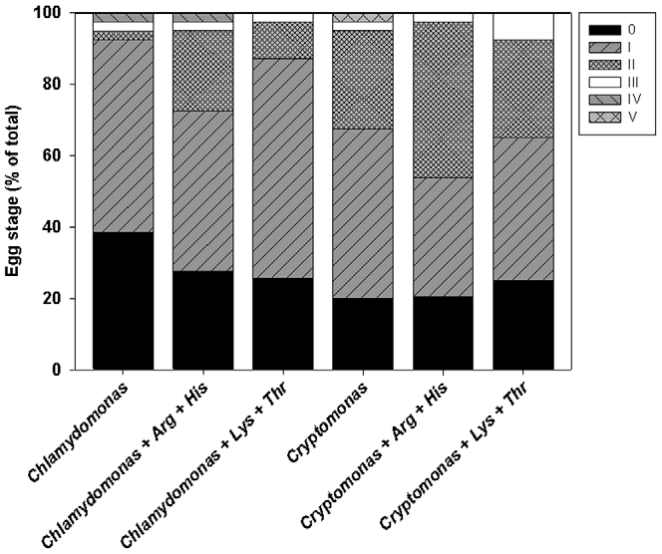
Development of subitaneous eggs. Developmental stage of *D. pulex* subitaneous eggs (% of total) depending on the food alga and the addition of dissolved EAA at concentrations of 25 µmol L^−1^; values given are means of n = 3 jars with 40 individuals each after 6 days of juvenile and pre-adult growth.

**Figure 5:**
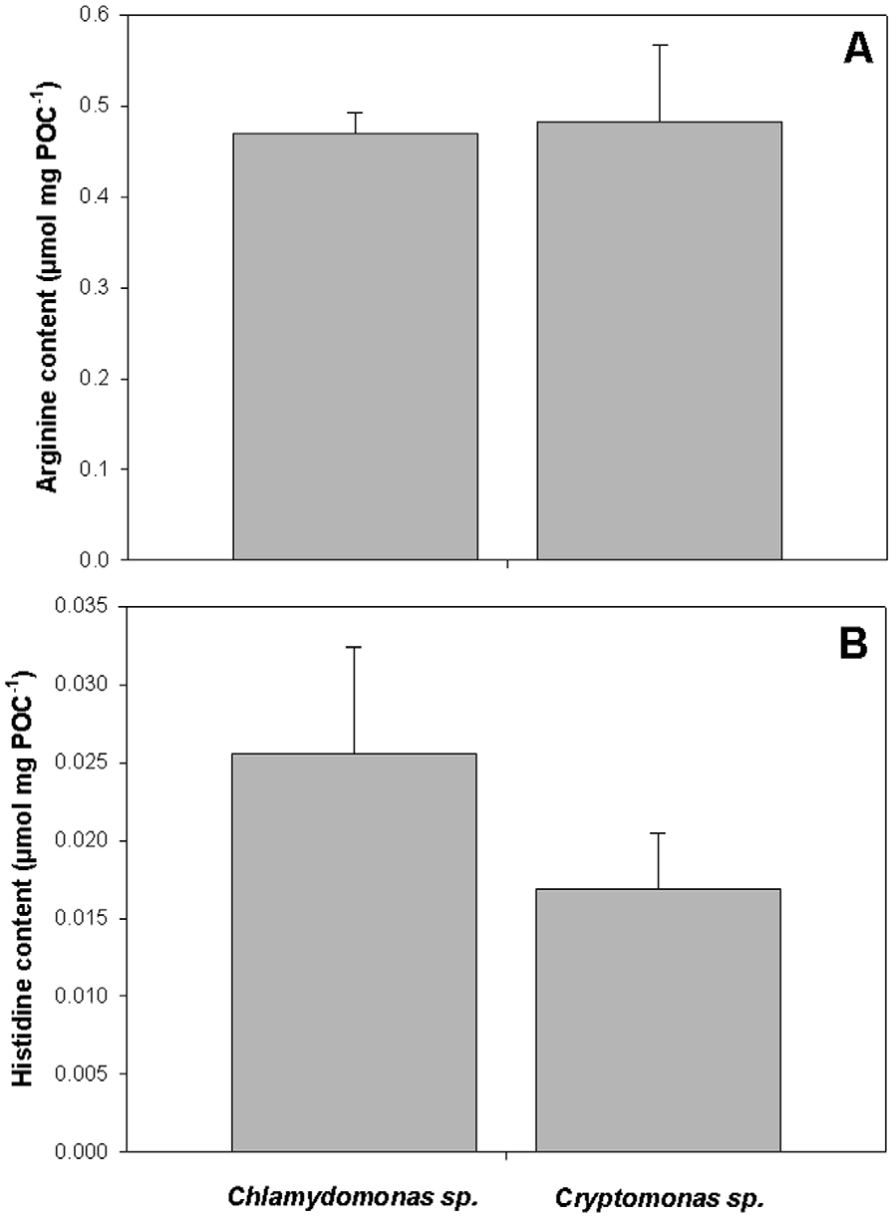
Amino acid content of algae. Arginine (A) and histidine (B) content of *Chlamydomonas* and *Cryptomonas* per mg of particulate organic carbon (n = 3); no significant differences were found using a one-way ANOVA; note the different scaling of plots A and B.

In a third reproduction experiment, we investigated the developmental stage of the daphnids' first clutch of eggs after six days of juvenile and pre-adult growth. Both feeding on *Cryptomonas* as well as *Chlamydomonas* with added Arg and His led to a higher frequency of advanced egg stages (≥ II according to [30]) at the end of the experiment in comparison to daphnids fed either pure *Chlamydomonas* or *Chlamydomonas* with the addition of Lys and Thr (Fig. 4): More than a quarter (mean ± SD of n = 4 independent replicates with 10 ind. each) of the mothers carried eggs that had reached an egg stage ≥ II after six days on a diet of *Cryptomonas* (32.5±15.0%) or *Chlamydomonas* with Arg and His (28.2±19.5%) while only a few of the eggs had reached this developmental stage on a diet of pure *Chlamydomonas* (7.7±9.7%) or of *Chlamydomonas* with Lys and Thr (12.8±9.5%, Fig. 5). Addition of Arg and His to *Cryptomonas* further increased the ratio of early developing eggs to 46.2±13.9% at a stage ≥ II. Almost all of the animals fed *Chlamydomonas* (92.3±9.7%) or *Chlamydomonas* with Lys and Thr (87.2±9.5%) either had no eggs in their brood pouch after six days or eggs in the first stage of development (Fig. 4). A one-way ANOVA on the relative amount of eggs in early (0-I) vs. later (≥ II) developmental stages between treatments revealed that there were significantly more eggs in a developmental stage ≥ II in the treatment with *Cryptomonas* with added Arg and His compared to the treatments with *Chlamydomonas* without added EAA or with added Lys and Thr (F_5,23_ = 4.413, p<0.01), but not when compared to the treatment with *Chlamydomonas* with added Arg and His (p = 0.458, Fig. 4).

### Amino acid analyses of microalgae

The Arg and His content of *Chlamydomonas* and *Cryptomonas* did not differ between species, and both algae contained approx. 10fold more Arg than His (Fig. 5). The His content of *Chlamydomonas* was even slightly (albeit not significantly) higher than that of *Cryptomonas* (Fig. 5). Not only the amino acid content, but also the protein content (Fink & von Berlepsch, unpubl. data) and C:N ratio of both algae was almost identical and not significantly different (one-way ANOVA, F_1, 3_ = 0.997, p = 0.39). The molar C:N ratios (5.3 and 5.1 for *Chlamydomonas* and *Cryptomonas,* respectively) were all well below the Redfield ratio of 6.6 [32], which allows us to exclude a nitrogen limitation of the algal cultures.

## Discussion

Since the seminal work of Schindler and co-workers [33], limnologists have considered phosphorus to be the major limiting nutrient in freshwater ecosystems and nitrogen has been considered to be of minor importance. However, only recently, studies have indicated that nitrogen limitation of freshwater primary production might be more common than previously thought [34], [35] and that the growth of freshwater herbivores can be limited by low dietary nitrogen availability [36], [37]. Furthermore, theoretical considerations by Anderson et al. [22] suggested that EAA availability has the potential to constrain the fitness of herbivores across ecosystems, independent of the total nitrogen availability. Numerous investigations related to aquaculture have provided evidence that both the growth and reproduction (yolk-provisoning of eggs) of fish and crustaceans require high amounts of EAA [27], [28], [38], [39], [40], [41]. Since EAA cannot be synthesized by consumers but have to be taken up with the diet, an imbalanced supply of EAA in the diet potentially limits the fitness of consumers, in particular for organisms that can not actively select for high quality food such as filter-feeders like *Daphnia*.

### EAA content of microalgae

To date, there is only very little known about the EAA content of planktonic algae. One of the few studies on the amino acid content of freshwater phytoplankton reported no differences in the (E)AA content between green algae (including the genus *Chlamydomonas* used in this study) and cryptophytes (*Rhodomonas* sp., Ahlgren et al. 1992). In this study, we found no differences in the arginine and histidine content of the two investigated algal cultures normalized to carbon, thus corroborating the findings of Ahlgren et al. [42]. From the viewpoint of algal physiology, this seems plausible, since probably all algal species co-occurring in the plankton of freshwater lakes have to synthesize a largely similar set of proteins for photosynthesis and general metabolism of unicellular autotrophs. Hence, the similar arginine and histidine content of *Chlamydomonas* and *Cryptomonas* cannot explain why *D. pulex* produced high numbers of resting eggs on a diet of *Chlamydomonas,* but not on *Cryptomonas*. Neither does it provide a clear explanation why the resting egg production of *D. pulex* was suppressed by the addition of these two EAA to a *Chlamydomonas* diet.

### EAA effects on somatic growth

The same set of amino acids appears to be essential for all classes of animals, ranging from vertebrates to arthropods, including the crustacean genus *Daphnia* [25]. EAA have multiple important functions in crustaceans, in particular for development [43], and production of peptide hormones [44], [45]. Unfortunately, there is only very limited data available on the EAA content and requirements of daphnids. Gardener and Miller [26] described the free amino acid pool in *D. magna* to be dominated by histidine, arginine, alanine, and glutamate. However, total amino acids in *D. magna* were not dominated by one specific amino acid, probably because they are all equally abundant in multiple proteins in the daphnids' body tissue. It is known that the growth of the marine decapod *Penaeus monodon* (which feeds primarily on phytoplankton and animal carrion) is positively correlated with the diet's content of the EAA arginine, histidine, leucine, isoleucine, phenylalanine and tryptophane [27], [39]. For the growth of the related species *Parapenaeus longirostris*, not only arginine, but also histidine was found to be particularly important [28]. Here, we did not find any evidence for a growth-promoting effect of a single EAA on *D. pulex*. Nevertheless it is possible, that similar to *P. monodon*, a specific combination and relative ratio of different EAA in the diet is needed to provide a higher growth rate of *Daphnia* sp. [39]. Addition of histidine in higher concentrations even had a negative effect on the growth of *D. magna* (Fink & Hohnsen, unpubl. data). Similarly, high concentrations of histidine caused negative effects on the growth of *P. monodon* [39] which might indicate a general intolerance of crustaceans to high concentrations of histidine. This EAA was reported to lead to necrosis in epithelial cells of the midgut gland of shrimps at high dietary concentrations [39]. However, without further physiological investigations we cannot conclude whether this mechanism was also responsible for the growth depression of *Daphnia* observed at high dietary histidine concentrations in our experiment.

### EAA effects on reproduction

Adult daphnids invest only minor proportions of their assimilated resources into somatic growth. Most of the assimilated carbon is invested in reproductive tissues. Nevertheless, they face a trade-off between the production of one or two, slowly developing resting eggs (as a strategy to overcome periods of unfavourable environmental conditions) and the production of up to 40 subitaneous eggs per clutch, which allows high population growth rates under sufficient resource availability and moderate mortality. Here, we demonstrate that the availability of the EAAs arginine and histidine, can modulate the switching between these alternative resource allocation strategies. This effect appears to be specific for arginine and histidine, as no switch in reproductive strategy was observed upon the addition of the EAAs lysine and threonine to a diet of *Chlamydomonas*. Since *Chlamydomonas* and *Cryptomonas* did not differ in their arginine and histidine content (Fig. 5), the suppression of resting egg production on *Chlamydomonas* by the addition of these two EAAs is not easy to explain. One possibility is that these EAAs exert an indirect effect on a yet unknown ephippia-inducing factor present in *Chlamydomonas*. However, due to the scarcity of literature on the molecular physiology of EAA uptake and metabolism in zooplankton, this remains speculative and clearly needs further studies. To date, the induction of resting eggs was considered to depend on photoperiod, population density and chemical cues (e.g, [9]). Only recently, Abrusan et al. [17] demonstrated that the diet's content in essential polyunsaturated fatty acids also plays an important role. The mechanism described here is an entirely new one and could possibly explain observations by Koch et al. [3] that addition of protein beads was able to suppress the resting egg formation of *D. pulex* on a green algal diet. Hence, the contribution of EAAs to the switching between reproductive strategies is a novel and to date overlooked interaction that could potentially affect population dynamics of *Daphnia*.

Further, the addition of these EAAs also increased the production of subitaneous eggs at saturating food quantities of both food organisms, thus demonstrating a clear food quality effect mediated through the EAA addition. This is particularly interesting since *Cryptomonas* is commonly assumed to be the optimal diet for *Daphnia* culture, consistently yielding high somatic growth and parthenogenetic reproduction rates (e.g., [46], [47]). Nevertheless, the rate of subitaneous egg production appears to be sub-maximal on (saturating quantities of) a pure *Cryptomonas* diet, since addition of arginine and histidine caused a significant increase in the number of subitaneous eggs, even on this high quality diet. The dissolved EAA were either directly taken up by *D. pulex* or attached to the algal cells and were ingested by the daphnids as particle-bound EAA. Both these uptake mechanisms might operate simultaneously, since the uptake of dissolved EAA was already shown for both planktonic crustaceans [26], [48] and green algae [49].

Dietary amino acids probably have further effects on the reproduction of *D. pulex*. We found the developmental stage of the subitaneous eggs (after six days) to depend on the addition of dissolved EAA: A higher percentage of subitaneous eggs in advanced developmental stages in the brood chambers of *D. pulex* fed a diet enriched in arginine and histidine suggests that these EAA influence not only the number, but also the development of subitaneous eggs. It is known that dietary EAAs are essential for the production and hatching success of subitaneous eggs of marine copepods [50], [51] and for the early growth phases of fish larvae [40], [41]. While the physiological background of EAA requirements has ben subject to extensive studies in fish [38], knowledge on the cellular and molecular effects of EAA in zooplankton is scarce. Here, we show that EAA availability might also influence the production (i.e. the timing of the release of eggs from the ovary into the brood chamber) or the actual embryonic development of cladoceran eggs. However, with the data available, we cannot distinguish which of these two mechanisms operates and we do not yet know how this would impact the total reproductive output of *D. magna*. This should be tested in future studies. Feeding on *Cryptomonas* produced similar egg development rates than the EAA-enriched green algal diet. This indicates that EAA-mediated nutritional quality can have additional effects on population growth rates via direct or indirect influences on the embryonic development at high maternal food quality conditions, although population growth rates were not determined in this experiment.

### Potential ecosystem effects of EAA availability

This is the first report to describe the effect of the availability of specific EAAs on the production of subitaneous and resting eggs in freshwater zooplankton, which could potentially have strong implications for freshwater food web dynamics. *Daphnia* are known to produce resting eggs at periods of high population density (crowding) and food shortage, two phenomena that are often highly correlated as an effect of the high community clearance rates of *Daphnia* sp. in the so-called „clear water phase“ in temperate lakes in spring or early summer. Nevertheless, the onset of resting egg production appears to be not only determined by crowding and food availability [9] and dietary fatty acids [17], but also by certain dietary amino acids. This novel and so far overlooked role of essential amino acids could potentially affect population dynamics of the freshwater keystone taxon *Daphnia*, which certainly advocates further studies.
