# Correction: Dietary Essential Amino Acids Affect the Reproduction of the Keystone Herbivore *Daphnia pulex*

**DOI:** 10.1371/annotation/6d71b282-8e08-43ba-bef7-7ad45cd48784

**Published:** 2012-06-15

**Authors:** Patrick Fink, Claudia Pflitsch, Kay Marin

This correction is issued to acknowledge the previous work by Dr Ulrike Koch and her colleagues which had stimulated our research and influenced our experimental approach but was unfortunately omitted from the list of cited references.

In her PhD thesis, Dr. Koch investigated food quality effects on and clonal differences in resting egg production of *Daphnia* sp. (Koch 2009). In a part of her thesis, she addressed the potential role of single amino acids for the reproductive mode of *Daphnia*. This work has stimulated our subsequent research presented in our paper (Fink et al. 2011). In her studies, Dr. Koch could demonstrate that the addition of specific dissolved amino acids (namely arginine and histidine) to a diet of the green alga *Scenedesmus* sp. (which together with crowding conditions induced resting egg formation in *Daphnia*, Koch 2009) triggered a change in *Daphnia*’s reproductive strategy from the production of resting eggs to the production of subitaneously developing eggs; this change also increased the population growth rate of daphnids (Koch et al. 2011). In our study using a similar experimental setup, we corroborated several of Dr. Koch’s findings, but also have extended her findings as well as reported some striking differences.

Using a different green algal genus (*Chlamydomonas*), we could confirm that addition of the amino acids arginine and histidine to a green algal diet induces a switch from resting egg production to the development of subitaneous eggs under crowding conditions. Interestingly, Koch (2009) has reported quite different effects of the two green algae *Scenedesmus* and *Chlamydomonas* on the reproduction of daphnids in her thesis: While she found daphnids to produce mostly subitaneous eggs on a diet of *Chlamydomonas*, but mostly resting eggs on a diet of *Scenedesmus* (under crowding conditions), our results suggest that also *Chlamydomonas* as a food organism leads to an increased resting egg production of daphnids compared to a high quality cryptophyte diet. Because of the effects of amino acid addition on the reproduction mode of *Daphnia*, Koch et al. (2011) investigated the effect of amino acid additions on the daphnids’ population growth rate. In our study, we followed another, independent hypothesis that the amino acid addition could also affect somatic growth of *Daphnia*. In an extension to the investigations of Koch et al. (2011), we further have demonstrated that the addition of arginine and histidine also influenced either the the timing of the release of eggs from the ovary into the brood chamber or the actual embryonic development of cladoceran eggs.

The most striking differences in the results of Dr. Koch and our study lie in the reported relative difference of the amino acid contents in the hydrolysates of the food algae. Koch (2009) and Koch et al. (2011) related the effects of algal diets on *Daphnia* reproduction to these algae’s amino acid contents. In contrast to their findings, we could not detect significant differences in the arginine and histidine contents of the algal diets in our experiment, despite their strongly different effects on the daphnids’ reproductive strategy (Fink et al. 2011). This corroborates earlier work by Ahlgren et al. (1992) who found no taxon-specific amino acid profiles in numerous phytoplankton taxa. Hence, our data suggests that the algal amino acid composition cannot explain the observed effects of algal diet on the reproductive strategy of *Daphnia* (Fink et al. 2011). These partially contradicting results on the content of essential amino acids of freshwater phytoplankton species and their potential consequences for herbivorous zooplankton remain to be resolved by future studies.

Taken together, the results of Koch and colleagues (2009, 2011) and our findings (Fink et al. 2011) indicate a high, but previously overlooked importance of specific essential amino acids for the reproductive strategy of the aquatic keystone herbivore *Daphnia*. Unfortunately, the citation of the work of Dr. Koch was accidentally deleted from a previous version of our manuscript during the assembly of our paper and was therefore unintentionally not included in the paper.

These references, for the omitted acknowledgements, were not listed in the published References list:

52.Koch U (2009) Resting egg production in *Daphnia*: food quality effects and clonal differences. PhD thesis. Limnological Institute, University of Konstanz, Germany

53.Koch U, Martin-Creuzburg D, Grossart HP, Straile D. (2011) Single dietary amino acids control resting egg production and affect population growth of a key freshwater herbivore. Oecologia 167 (4): 981-989. DOI: 10.1007/s00442-011-2047-4 

